# Muscle Eccentric Contractions Increase in Downhill and High-Grade Uphill Walking

**DOI:** 10.3389/fbioe.2020.573666

**Published:** 2020-10-14

**Authors:** Xiao Hu, Nathaniel T. Pickle, Alena M. Grabowski, Anne K. Silverman, Silvia S. Blemker

**Affiliations:** ^1^Department of Biomedical Engineering, University of Virginia, Charlottesville, VA, United States; ^2^Department of Mechanical Engineering, Colorado School of Mines, Golden, CO, United States; ^3^Department of Integrative Physiology, University of Colorado Boulder, Boulder, CO, United States; ^4^Department of Veterans Affairs, VA Eastern Colorado Healthcare System, Denver, CO, United States; ^5^Department of Orthopedic Surgery, University of Virginia, Charlottesville, VA, United States; ^6^Department of Mechanical and Aerospace Engineering, University of Virginia, Charlottesville, VA, United States

**Keywords:** musculoskeletal simulation, sloped walking, Duchenne muscular dystrophy, eccentric contraction, muscle coactivation

## Abstract

In Duchenne muscular dystrophy (DMD), one of the most severe and frequent genetic diseases in humans, dystrophic muscles are prone to damage caused by mechanical stresses during eccentric contractions. Eccentric contraction during walking on level ground likely contributes to the progression of degeneration in lower limb muscles. However, little is known about how the amount of muscle eccentric contractions is affected by uphill/downhill sloped walking, which is often encountered in patients’ daily lives and poses different biomechanical demands than level walking. By recreating the dynamic musculoskeletal simulations of downhill (−9°, −6°, and −3°), uphill (+3°, +6°, and +9°) and level walking (0°) from a published study of healthy participants, negative muscle mechanical work, as a measure of eccentric contraction, of 35 lower limb muscles was quantified and compared. Our results indicated that downhill walking overall induced more (32% at −9°, 19% at −6°, and 13% at −3°) eccentric contractions in lower limb muscles compared to level walking. In contrast, uphill walking led to eccentric contractions similar to level walking at low grades (+3° and +6°), but 17% more eccentric contraction at high grades (+9°). The changes of muscle eccentric contraction were largely predicted by the changes in both joint negative work and muscle coactivation in sloped walking. As muscle eccentric contractions play a critical role in the disease progression in DMD, this study provides an important baseline for future studies to safely improve rehabilitation strategies and exercise management for patients with DMD and other similar conditions.

## Introduction

Duchenne muscular dystrophy (DMD) is one of the most severe and frequent genetic diseases in humans, affecting 1 in 3500 male births (Mah et al., 2014). In DMD, the absence of the dystrophin protein due to genetic mutation makes muscle prone to damage caused by mechanical stresses during contractions, especially eccentric contractions (Petrof et al., 1993; Childers et al., 2002). Although the repeated damage to muscle leads to progressive degeneration, muscle degeneration in DMD progresses differently across muscles (Arpan et al., 2013; Willcocks et al., 2014; Wokke et al., 2014; Li et al., 2015). Specifically, this selective degeneration in lower limb muscles is likely related to the varying amount of eccentric contractions from muscles in walking on level ground (Hu and Blemker, 2015; Hu et al., 2017). In addition to level walking, when individuals with DMD (mostly children) are exploring the world and interacting with their peers, they will encounter various activities in their daily living, such as uphill/downhill walking, ascending/descending stairs, running, jumping, etc. However, changes in eccentric contractions in different types of activities in the general population as well as patients with DMD remain unclear. This knowledge will help provide scientific guidance for a safer and more effective care, rehabilitation and exercise management for patients with DMD, thereby improving their quality of life (Markert et al., 2012; Birnkrant et al., 2018; Spaulding and Selsby, 2018). Before studying various activities in patients with DMD, it is critical to establish a baseline understanding of muscle eccentric contractions in healthy participants. This understanding could help estimate risk-to-benefit ratio of certain activities and provide data-driven hypotheses for future studies to determine the relative detriment and/or benefit of a given activity for patients with DMD. Therefore, we investigated how uphill/downhill sloped walking, which is essential in daily living, affects the amount of muscle eccentric contractions compared to level walking in healthy participants, aiming to provide a foundation for future studies on sloped walking in patients with DMD.

Many previous studies have demonstrated that sloped walking imposes unique kinematic and kinetic demands on the lower limbs compared to level walking. In kinematics, uphill walking generally leads to greater flexion at all three joints of the lower limb around the point of heel strike and greater extension of all three joints during midstance to raise the limb for toe clearance and propel the body up the slope (Lay et al., 2006; Dewolf et al., 2018). In contrast, during downhill walking, the knee flexes more during the stance phase to lower the body down the slope (Lay et al., 2006; Dewolf et al., 2018). These differences lead to changes in the sagittal plane ranges of motion of all three lower limb joints (Lange et al., 1996; Kimel-Naor et al., 2017; Montgomery and Grabowski, 2018). In joint kinetics, sloped walking induces greater peak joint moments and joint compression forces in the hip, knee and ankle joints compared to level walking (Redfern and Dipasquale, 1997; Lay et al., 2006; Alexander and Schwameder, 2016c). In addition, to raise/lower the body center of mass, the lower limb generates progressively increased positive/negative work as the grades of uphill/downhill walking increase (DeVita et al., 2007; Franz et al., 2012; Alexander et al., 2017). Specifically, of the lower limb joints, the ankle joint generates the most positive work in uphill walking, whereas the knee joint generates the most negative work in downhill walking (Alexander et al., 2017; Montgomery and Grabowski, 2018). While these results provide a general picture of how mechanical work at the joint and whole limb levels varies in sloped walking, it remains unclear how muscle work may vary in sloped walking, as there are elevated coactivations in muscles in sloped walking, especially in uphill walking (Lay et al., 2007; Franz and Kram, 2013).

Few studies have specifically examined how sloped walking influences the actions of individual muscles. In experimental studies, the measured electromyographical (EMG) signals have indicated greater activities in hip, knee and ankle extensors in uphill walking compared to level walking (Lay et al., 2007; Franz and Kram, 2012). By contrast, in downhill walking, there were greater activities only in knee extensors compared to level walking (Lay et al., 2007; Franz and Kram, 2012). In addition, greater muscle coactivations were observed in uphill walking compared to level walking (Lay et al., 2007; Franz and Kram, 2013). Recently, simulations of musculoskeletal models demonstrated the ability to predict muscle activities and forces during sloped walking, and indicated substantial changes in lower limb muscle activities, forces and the associated joint contact forces compared to level walking (Alexander and Schwameder, 2016a,b). By quantifying the power delivered to body segments (e.g., trunk, legs) by muscles, Pickle et al. (2016) demonstrated that hip extensors transfer more power from the trunk to legs (i.e., absorb power from trunk and deliver power to legs) in uphill walking compared to level walking, whereas rectus femoris (RF) transfers more power from the legs to the trunk in downhill walking than in level walking. Although these experimental and simulation studies have advanced our understanding of muscle function in sloped walking, the extent to which the amount of eccentric contractions of individual lower limb muscles changes in sloped walking compared to level walking remains unanswered.

The goals of this study were to examine the amount of eccentric contraction in lower limb muscles during sloped walking and compare them with those in level walking in healthy participants. During eccentric contractions, muscle is actively generating contraction force while being lengthened in the opposite direction, resulting in the production of negative work. Therefore, calculation of negative work done by a muscle can be used to quantify the amount of eccentric contractions of muscle during walking. As lower limb joints generate more negative work in downhill walking than in level walking (Franz et al., 2012; Alexander et al., 2017; Montgomery and Grabowski, 2018), we hypothesized that lower limb muscles generate greater amount of negative work in downhill walking, suggesting greater amount of eccentric contractions. As lower limb joints generally generate less negative work in uphill walking than level walking (Franz et al., 2012; Alexander et al., 2017; Montgomery and Grabowski, 2018), we hypothesized that lower limb muscles would have reduced negative work in uphill walking. To test these hypotheses, musculoskeletal simulations of downhill, level and uphill walking of healthy participants from a previous study (Pickle et al., 2016) were recreated to estimate and compare the amount of eccentric contractions in 35 lower limb muscles (see Figure 2 for the list of 35 muscles) in these different walking conditions. These results will reveal how the amounts of muscle eccentric contractions vary in sloped walking compared to level walking and provide baseline data for future studies to establish scientific guidance for safely improving care, rehabilitation and exercise management for patients with DMD.

## Materials and Methods

### Calculation of Muscle and Joint Negative Work

The dynamic musculoskeletal simulations of sloped and level walking from a previous published study (Pickle et al., 2016) were used as the basis to estimate the amount of negative work, a measure of eccentric contractions, in lower limb muscles. Briefly, in that study, a total of 273 dynamic simulations of 13 healthy participants (4 female/9 male, mean ± standard deviation: 67 ± 10 kg, 173 ± 9 cm, 28 ± 7 years) walking on an instrumented treadmill were developed based on a lower limb musculoskeletal model with 21 degrees-of-freedom and 92 Hill-type muscle-tendon units (Delp et al., 1990). These simulations included walking at 1.25 m/s on three downhill (−9°, −6° and −3°) and uphill (+3°, +6°, and +9°) slopes as well as level ground (0°) with three gait cycles for each slope. All 273 simulations were recreated using the Muscle Analysis in the Analyze Tool in OpenSim 3.3 (Delp et al., 2007). The time histories of the outputs from the original simulations that described the model states and muscle excitations were input to the Muscle Analyses to recreate each simulation as it occurred in the original study and then generate data describing all attributes of muscle behavior, including fiber lengths and velocities, fiber power due to active force, muscle moment arm.

Fiber power due to active force of muscle was used to estimate the amount of eccentric contraction of each muscle during walking. Specifically, negative fiber power (power < 0) was integrated over the duration of one gait cycle to calculate the negative work of for each of 35 lower limb muscles, which was used as a measure of the amount of eccentric contraction (Hu and Blemker, 2015). Only the power caused by active muscle force was considered, because active lengthening of muscle induces much more damage to dystrophic muscles compared to passive lengthening (Petrof et al., 1993). To account for the structural variation of fiber lengths and cross-sectional areas among muscles, negative work was normalized by the optimal fiber length and maximum isometric force of each muscle. For a muscle represented by multiple muscle-tendon units, the average value across these muscle-tendon units was used. The total normalized negative work of all lower limb muscles was calculated by summing the normalized negative work of individual muscles to understand how overall muscle negative work varied in sloped walking.

Joint powers due to the net flexion/extension moments at the hip, knee and ankle joints were also calculated from the simulations (Pickle et al., 2016). Similar to muscles, the negative joint power was integrated over the duration of one gait cycle to estimate the negative joint work of the hip, knee and ankle flexion/extension during sloped and level walking. The estimated joint work was then normalized by each participant’s mass for analyses across all participants. The total normalized negative work of all joints was the sum of the normalized negative work from hip, knee and ankle joints. Note that only the amounts (i.e., magnitudes) of muscle/joint negative work were compared in the following analyses.

### Calculation of Muscle Coactivation

Previous studies have observed greater muscle coactivation during sloped walking (Lay et al., 2007; Franz and Kram, 2013), which may affect the amounts of muscle eccentric contractions. Therefore, the levels of muscle coactivations were assessed using moment-based coactivation indices (CI) for the multiple muscles across the hip, knee and ankle joints (Le et al., 2017). The flexion/extension muscle moments in one gait cycle for the muscles across the hip, knee and ankle were calculated as the product of the active force and the moment arm of one muscle with respect to the corresponding joint. The net joint moment was the sum of the moments of all muscles with respect to a given joint. Moments in the hip flexion, knee flexion and ankle plantarflexion directions were defined positively. At each time step, muscles were classified as agonists if they generated moments with the same sign as the net joint moment, and antagonists if they generated moments with the opposite sign of the net joint moment. The ratio of antagonists to agonists (*b*(*t*)) was defined as:

(1) $$b\,\left(t\right)=\frac{\sum_{j}{\text{antagonist}}_{j}\,\left(t\right)}{\sum_{i}{\text{agonist}}_{i}\,\left(t\right)}$$

where agonist_*i*_ (*t*) is magnitude of the moment of the *i*th agonist, and antagonist_*j*_ (*t*) is the magnitude of the moment of the *j*th antagonist. The total absolute moments of agonists and antagonists across one joint (*M*_*total*_) were calculated as:

(2) $$M_{\text{total}}\,\left(t\right)=\sum_{i}{\text{agonist}}_{i}\,\left(t\right)+\sum_{j}{\text{antagonist}}_{j}\,\left(t\right)$$

No normalization was applied to the moments in Eqs 1 and 2. The moment-based coactivation index of a joint (*CI*_*jnt*_) was then defined as the ratio of antagonists to agonists (*b*(*t*)) multiplied by the total absolute moment (*M*_*total*_(*t*)) normalized by the maximum total absolute moment across all the trials and slopes for each participant (*M*_*max*_):

(3) $$C\,I_{\text{jnt}}\,\left(t\right)=b\,\left(t\right)*\left(\frac{M_{\text{total}}\,\left(t\right)}{M_{\text{max}}}\right)$$

The *M*_*max*_ was used so that *CI*_*jnt*_ could be compared across various walking conditions. The average of *CI*_*jnt*_ over the entire gait cycle was calculated to represent the level of coactivation at a joint during walking.

### Statistical Analysis

Linear mixed-effects models (Winter, 2013) with slopes as a fixed effect and participants as a random effect were used to examine the effects of slopes on muscle or joint functions during sloped walking compared to level walking. For each of the 35 lower limb muscles (Figure 2), if the ANOVA had a significant slope fixed effect, the amount of muscle negative work (dependent variable) were assessed between each grade of sloped walking (downhill: −9°, −6° and −3°; uphill: +3°, +6°, and +9°) and level walking (0°) using the sequential Bonferroni correction (Holm–Bonferroni method) to control multiple comparisons (Holm, 1979). Similarly, the fixed effect of slope on (1) total normalized negative work of all lower limb muscles, (2) total normalized negative work of all joints, and (3) coactivation of muscles across the hip, knee and ankle joints were examined with participants as a random effect.

To further investigate the underlying factors that contributed to the changes of muscle negative work in sloped walking, a linear mixed-effects model with total joint negative work and coactivation at the hip as two fixed effects (predictor variables), participants as a random effect and total muscle negative work as the dependent variable was used, referred to later as the full model. The full model assessed whether each of two predictors was significant in predicting total muscle negative work. In addition, linear mixed-effects models resulted from dropping either the total joint negative work or the coactivation at the hip were also constructed, referred to later as the reduced models. The variances of the total muscle negative work explained by the full and the reduced models were then calculated and compared to assess whether each predictor substantially improved the full model’s ability to describe the changes in total muscle negative work in sloped walking. All statistical analyses were conducted in Matlab (R2018b, Mathworks, Natick, MA, United States) with the significance level set at *p* < 0.05.

## Results

### Muscle Negative Work in Downhill Walking

**FIGURE 1:**
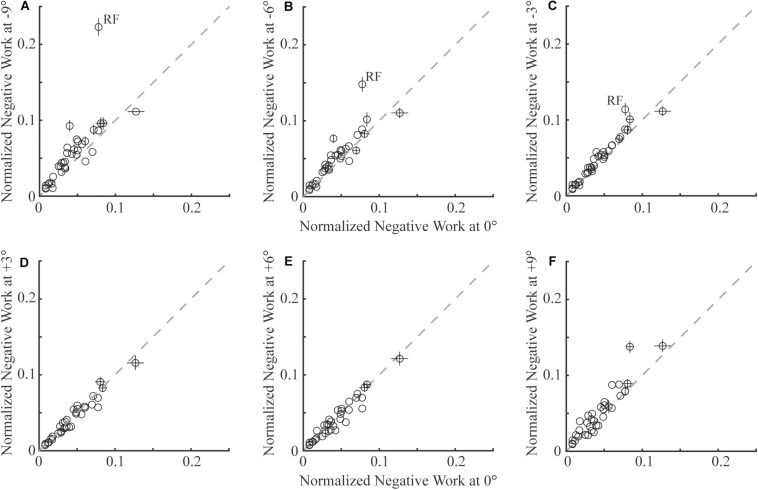
Comparison of negative work of 35 lower limb muscles (each circle represents the mean and standard error across participants of one muscle) in sloped walking and in level walking. Panels **(A–C)** are comparisons between downhill walking at –9°, –6°, and –3° and level walking at (0°), respectively. Panels **(D–F)** are comparisons between uphill walking at +3°, +6°, and +9° and level walking at 0°, respectively. In all panels, *x*-axes are negative work in level walking, and *y*-axes are negative work in sloped walking at various grades. The dashed line is the unity line, indicating equal amounts of negative work in sloped and level walking. Error bars smaller than circles are not shown. RF: rectus femoris muscle.

**FIGURE 2:**
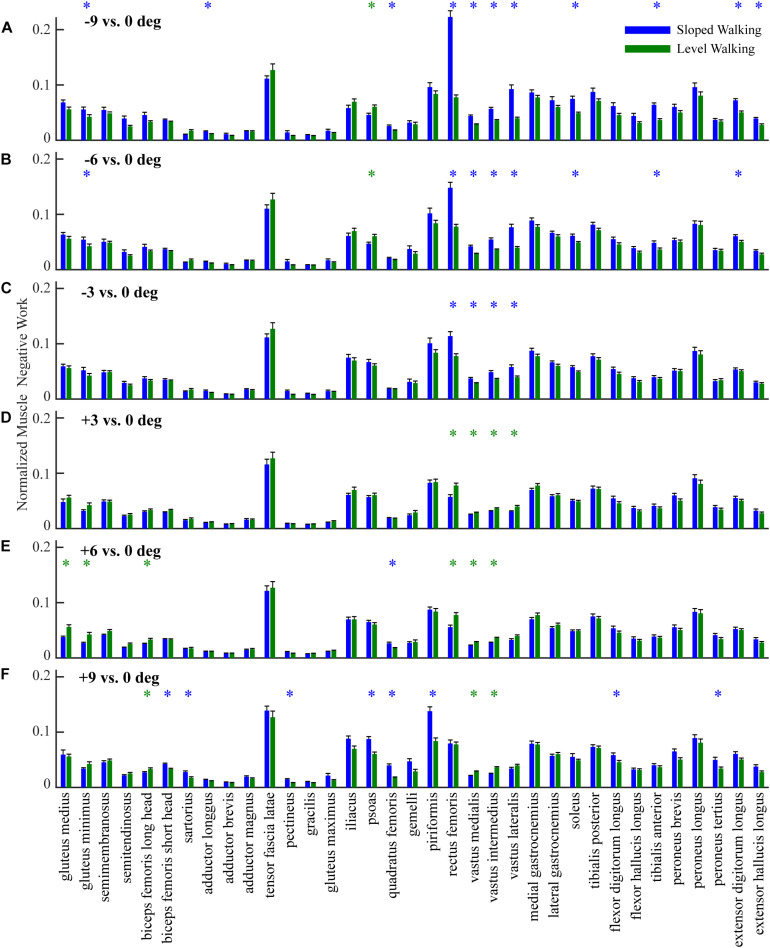
Comparison of negative work of 35 lower limb muscles (mean and standard error across participants) in sloped walking and in level walking. Panels **(A–C)** are comparisons between downhill walking at –9°, –6°, and –3° and level walking at 0°, respectively. Panels **(D–F)** are comparisons between uphill walking at +3°, +6°, and +9° and level walking at 0°, respectively. Blue/green ‘*’ indicates negative work is significantly higher/lower in sloped walking compared to level walking (*p* < 0.05).

**FIGURE 3:**
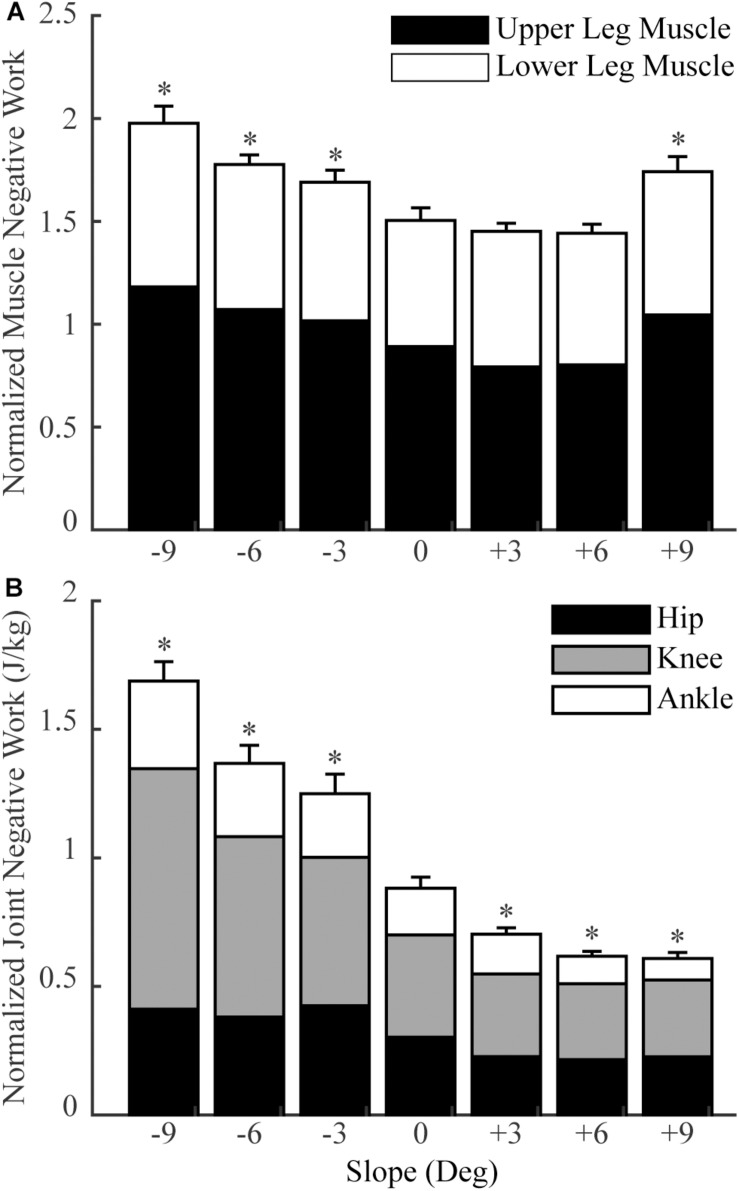
The total normalized muscle negative work **(A)** and total normalized joint negative work **(B)** in sloped and level walking (mean and standard error across participants). Upper leg muscles: muscles that cross the hip and knee joints (no gastrocnemius); lower leg muscles: muscles that cross the ankle joint (including gastrocnemius). ‘*’ = significant difference from level walking (*p* < 0.05).

In general, lower limb muscles performed larger amounts of negative work in downhill walking compared to level walking. In downhill walking at −9°, −6°, and −3°, 11, 8, and 4 muscles had significantly more negative work compared to level walking (0°), respectively (Figures 1A–C, 2A–C). The negative work of these muscles was 69 ± 13%, 48 ± 8% and 39 ± 2% (mean ± standard error) more in downhill walking at −9°, −6°, and −3°compared to level walking, indicating larger amounts of eccentric contraction. In contrast, only the psoas muscle performed significantly less negative work at −9° and −6° than in level walking. As a result, the amount of total muscle negative work (sum of negative work across all lower limb muscles) significantly increased by 32 ± 5%, 19 ± 4%, and 13 ± 3% in downhill walking at −9°, −6°, and −3°, respectively (*p* < 0.001 for −9°, −6°, −3°; Figure 3A), which was likely driven by the significant increases (*p* < 0.001 for −9°, −6°, −3°) of total negative joint work (sum of negative work across all lower limb joints; Figure 3B). Particularly, muscle negative work increased the most in the knee extensors, especially the RF, which was consistent with the large increases of knee joint negative work in downhill walking compared to level walking (Figure 3B, gray portion).

### Muscle Negative Work in Uphill Walking

In contrast to downhill walking, the amount of negative work of lower limb muscles in uphill walking at low grades (+3° and +6°) was similar to that in level walking (0°), but increased at large grades (+9°) compared to level walking (0°). In uphill walking at +3° and +6°, only four and six muscles, mostly the knee extensors (RF and vastus muscles), performed significantly less negative work than level walking at 0°, whereas there was also one muscle at +6° that had significantly more negative work than at 0° (Figures 1D,E, 2D,E). As a result, the amount of total negative work of all lower limb muscles was similar at +3° and +6°compared to 0° (+3°: *p* = 0.33; +6°: *p* = 0.25; Figure 3A). This muscle level result was different from the joint level, where the total amount of negative work of all lower limb joints was significantly lower at +3° and +6° than at 0° (*p* < 0.001 for +3°, +6°; Figure 3B).

When the inclination increased to +9°, a greater number of muscles performed larger amount of negative work at +9° than at 0°. There were eight muscles with significantly more negative work at +9°compared to 0°, whereas there were only three muscles with significantly less negative work at +9°compared to 0° (Figures 1F, 2F). Overall, the amount of total muscle negative work of all lower limb muscles significantly increased by 17 ± 5% at +9° than at 0° (*p* = 0.002), with 65% of the increase from upper leg muscles (Figure 3A). This increase of total muscle negative work at +9° differed from the total joint negative work, which remained significantly lower than 0° (*p* < 0.001), similar to +3° and +6° (Figure 3B).

### Muscle Coactivations in Sloped Walking

**FIGURE 4:**
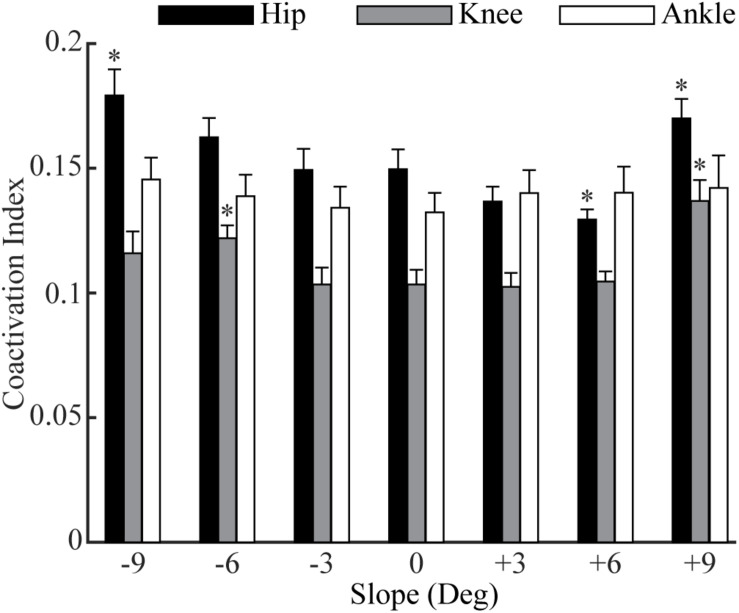
The moment-based coactivation indices (mean and standard error across participants) for the muscles across the hip, knee, and ankle joints in sloped and level walking. ‘*’ = significant difference from level walking (*p* < 0.05).

The overall coactivations at the hip, knee and ankle joints contributed from all muscles across these joints varied across slopes (Figure 4). In downhill walking, coactivation at the hip was significantly higher at −9° (*p* = 0.01), but there was no difference at −6° (*p* = 0.07) and −3° (*p* = 0.96) compared to 0°. In uphill walking, coactivation at the hip was comparable at +3° (*p* = 0.07), less at +6° (*p* = 0.004), but greater at +9° (*p* = 0.004) compared to 0°. For the knee, the coactivations at −9°, −3°, +3°, and +6° were comparable to 0°, but significantly increased at −6° (*p* < 0.001) and +9° (*p* < 0.001) compared to 0°. The coactivations of the ankle did not change significantly for any of the six slopes compared to level walking (*p* = 0.52).

### Factors Predicting the Changes of Total Muscle Negative Work

**FIGURE 5:**
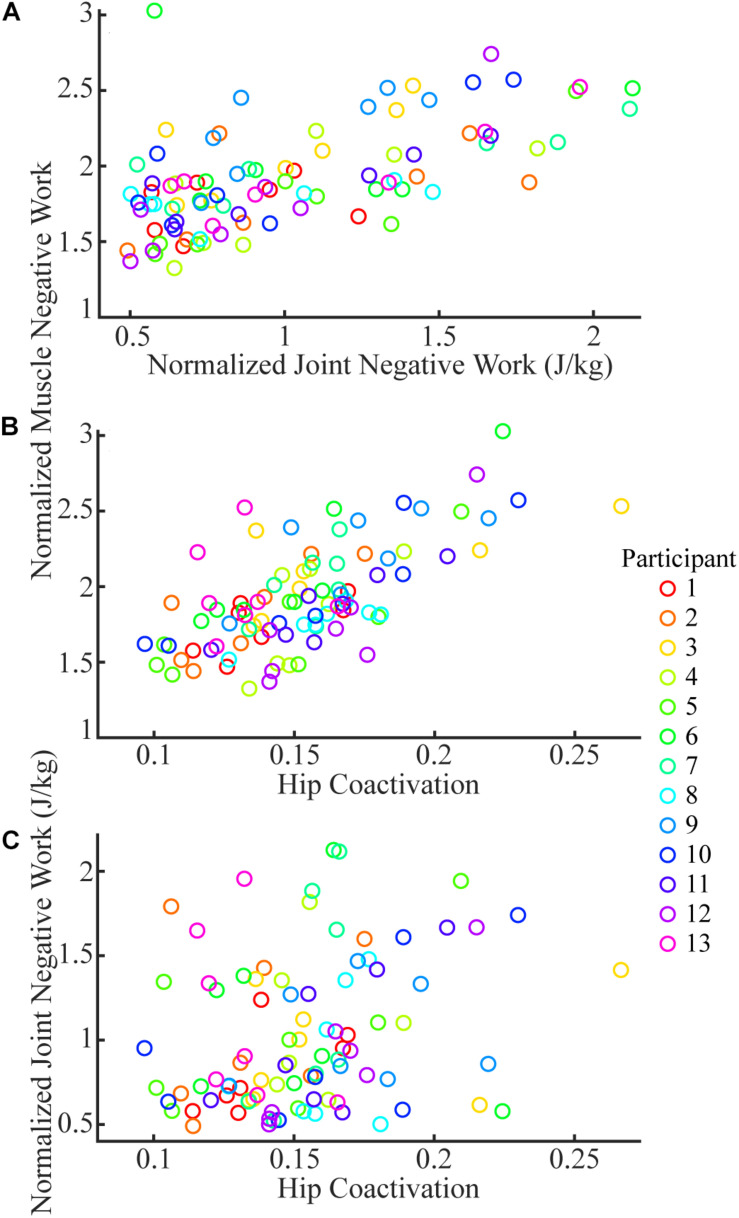
The joint negative work **(A)** and coactivation at the hip **(B)** were significant predictors for total muscle negative work. There was no meaningful relationship between coactivation at hip and joint negative work **(C)**. Each color represents one participant, and each circle is the mean of three trials of a given participant at a given slope.

The total joint negative work and the coactivation at the hip both significantly predicted the changes of total muscle negative work in sloped walking (Figures 5A,B). Fitting a linear mixed-effects model with total joint negative work and coactivation of hip flexion/extension as two predictors and total muscle negative work as the dependent variable indicated that both predictors had significant effects on the changes of the total muscle negative work (*p* < 0.001 for both predictors). The fitted full model accounted for 85% of the variance of the total muscle negative work across 13 participants and 7 slopes (i.e., *r*^2^ = 0.85). Dropping either the total joint negative work or the coactivation at the hip as a predictor substantially lowered the reduced model’s ability to explain the variance of the total muscle negative work to 62 or 49%, which further supported both joint negative work and coactivation at the hip as two significant predictors of the changes of total muscle negative work. There was no meaningful relationship between the joint negative work and the coactivation at the hip (Figure 5C), as fitting a linear mixed effect model between the two only resulted in an insignificant *r*^2^ value of 0.07.

## Discussion

The goals of this study were to estimate and then compare the amount of eccentric contraction in lower limb muscles during sloped walking with those in level walking. These goals were accomplished by recreating the dynamic musculoskeletal simulations of sloped and level walking from a previous study (Pickle et al., 2016) and then analyzing muscle negative work as a measure of eccentric contraction in various walking conditions (slopes at −9°, −6°, −3°, +3°, +6°, and +9°; level at 0°). Our results suggested that lower limb muscles generally performed a greater amount of eccentric contraction in downhill walking compared to level walking. The lower limb muscles overall had 32%, 19%, and 13% more eccentric contraction at −9°, −6°, and −3°compared to level walking, respectively. Our results also showed that although uphill walking at low grades (+3° and +6°) generally led to similar eccentric contraction in muscles, high grades (+9°) may induce greater amounts of eccentric contraction in certain muscles (Figure 2F) compared to level walking. The changes in muscle eccentric contraction with slope were largely predicted by the changes in both joint negative work and muscle coactivation. These results revealed how the amount of eccentric contraction that the lower limb muscles perform is affected by sloped walking and the related underlying biomechanical mechanisms. As muscle eccentric contractions play a critical role in the disease progression in DMD, this study provides important baseline data for future studies to safely improve rehabilitation strategies and exercise management for patients with DMD.

**FIGURE 6:**
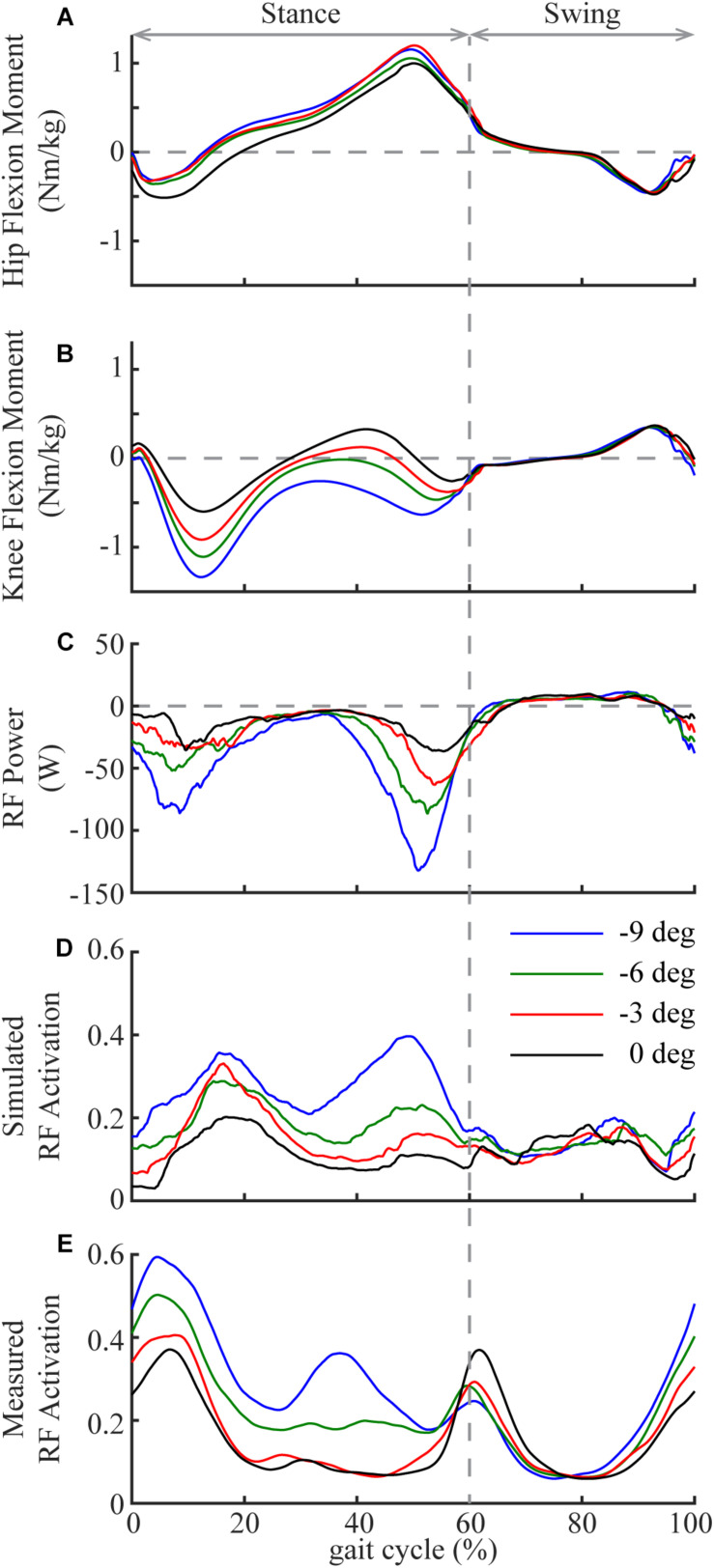
Mean hip (**A**; flexion is positive) and knee (**B**; flexion is positive) joint moments in one gait cycle (heel strike to heel strike) in downhill and level walking. Mean power **(C)**, simulated activation **(D)** and measured activation **(E)** of rectus femoris (RF) in one gait cycle in downhill and level walking. Note the substantial increase of activation and negative power of RF during the second half of the stance phase, which is likely due to the preferential activation of RF to simultaneously generate hip flexion and knee extension moments.

Among all the lower limb muscles, the knee extensors had the largest increases in eccentric contraction during downhill walking, primarily due to the changes in the mechanical demands of this task and the anatomical configuration. Although lower limb muscles generally had greater eccentric contraction in downhill walking compared to level walking, the knee extensors were most affected (Figures 1A–C, 2A–C). To lower the body center of the mass, the knee joint had greater extension moments and generated greater amount of negative work during a large portion of the stance phase during downhill walking compared to level walking (Kuster et al., 1995; Lay et al., 2006, 2007). These mechanical demands were achieved by the knee extensor muscles, as they performed the substantially increased eccentric contraction in downhill walking. Eccentric contraction increased the most in the RF (Figures 1A–C, 2A–C). As a biarticular muscle that generates knee extension and hip flexion moments, the drastic increase of eccentric contraction, as measured by the amount of negative work, in RF may be due to its preferential activation in the second half of stance (Prilutsky, 2000), in which simultaneous hip flexion and knee extension moments occurred in downhill walking (Figures 6A,B). The negative power and both the simulated and measured activations showed increased RF activities in this time period in downhill walking compared to level walking (Figures 6C–E), supporting the preferential activation of RF.

Our results suggested that the amount of negative work of lower limb muscles in sloped walking varied not only with the amount of the joint negative work but also with the muscle coactivations. Previous studies (DeVita et al., 2007; Franz et al., 2012; Alexander et al., 2017; Montgomery and Grabowski, 2018) and the current study (Figure 3B) all showed that the joint negative work decreased from downhill to level to uphill walking. As joint work is largely produced by muscles surrounding the joint, it is not surprising that the amount of muscle negative work had similar behavior to joint negative work, decreasing from downhill to level walking (Figure 3A). Our statistical analyses further corroborated that joint negative work was a significant predictor of the muscle negative work in sloped walking (Figure 5A). However, joint negative work, as typically calculated in previous studies (DeVita et al., 2007; Alexander et al., 2017; Montgomery and Grabowski, 2018), was only based on the net joint moments and joint angular velocities, which did not incorporate any potential influence from muscle coactivations. Muscle coactivations can lead to additional muscle negative work that is not reflected in net joint negative work. Consistent with previous observations (Lay et al., 2007; Franz and Kram, 2013), our analyses of muscle activations indicated greater coactivations in muscles crossing the hip joint during uphill walking, particularly at high grades (Figure 4), consistent with the increase of the amount of muscle negative work in uphill walking at +9° (Figures 1F, 2F). This led us to assess whether muscle coactivations at the hip joint may partially predict changes of total muscle negative work. Using linear mixed-effects models, we demonstrated that muscle coactivations at the hip joint are another significant predictor of the muscle negative work (Figure 5B), accounting for the variations in muscle negative work that could not be explained by joint negative work alone. Together, our analyses indicated that although joint negative work may have a direct effect on muscle eccentric contraction, greater muscle coactivations could also lead to increased muscle eccentric contraction. The greater muscle coactivations may be related to the greater demands for joint moments during sloped walking. Greater moment demands at one joint spanned by biarticular muscles in sloped walking may in turn require coactivation of antagonist muscles at the other joint spanned by the same biarticular muscles (Lay et al., 2007).

Altered kinematics in sloped walking may also contribute to the variation of muscle eccentric contraction. For example, the range of motion of the hip in the sagittal plane is larger in uphill walking than in level walking (Lay et al., 2006; Montgomery and Grabowski, 2018), which was also observed in the current study (Supplementary Figure 1A). This increased range of motion led to a higher lengthening velocity of the hip flexors (e.g., iliacus) during hip extension in the stance phase of uphill walking, which caused increased negative power and consequently negative work in uphill walking, (Supplementary Figures 1B,D). In addition, joint negative work changes with walking speed during sloped and level walking, with higher speeds generally leading to larger negative work (Farris and Sawicki, 2012; Montgomery and Grabowski, 2018). Although not specifically investigated in the current study, muscle eccentric contraction may be expected to increase with the speed during sloped and level walking. Given that patients with DMD tend to walk slower than typically developing children (Goudriaan et al., 2018), future studies may examine the extent to which a lower walking speed in patients with DMD may serve as a protective mechanism in preserving dystrophic muscles.

The knowledge of the influence of sloped walking on eccentric contractions of lower limb muscles in healthy participants obtained in this study may provide insightful directions for future studies that aim to improve the understanding and management of DMD. As demonstrated in this study, lower limb muscles generally experienced increased eccentric contractions during downhill walking compared to level walking. As joint negative work dictated by walking conditions strongly predicts the increase of muscle eccentric contraction (measured by muscle negative work) in downhill walking (see section “Factors Predicting the Changes of Total Muscle Negative Work”), it is plausible that the overall increase of muscle eccentric contraction may also occur in patients with DMD during downhill walking. However, the increase of eccentric contraction across lower limb muscles may be somewhat different from that shown in current study (Figure 2) due to potential differences in walking characteristics and muscle properties between the healthy population and patients with DMD (D’Angelo et al., 2009; Gaudreault et al., 2009; Thomas et al., 2010; Klingler et al., 2012; Wokke et al., 2014; Goudriaan et al., 2018). Therefore, future studies could examine the extent to which the downhill walking may exacerbate muscle eccentric contractions that lead to more damage to dystrophic muscles in patients with DMD compared to level walking. These studies will help determine whether downhill walking should be avoided in this population. However, precaution needs to be taken in these future studies as downhill walking may be associated with an unfavorable risk-to-benefit ratio. Our results also highlighted the knee extensors as the most adversely affected muscles in the downhill walking, which is consistent with imaging observations that these muscles are the most degenerated muscle group in DMD (Wokke et al., 2014). These results suggested a possible link between sloped walking and the selective muscle degeneration of DMD that is worth further investigation.

The influence of uphill walking on muscle eccentric contractions is complex. At low grades (e.g., +3° and +6° in current study), although lower limb muscles overall performed comparable eccentric contractions compared to level walking (Figure 3A), the knee extensors experienced smaller amounts of eccentric contractions (Figures 2D,E). In contrast, high-grade uphill walking (e.g., +9° in current study) resulted in greater amounts of eccentric contractions. Further studies are needed to determine whether these results hold in patients with DMD, and if so, the safe grades of inclination that may be suitable for exercise and the harmful grades of inclination that lead to greater amounts of eccentric contractions. Simulations, as demonstrated in the current study, can serve as a viable approach for future studies to quantify the amount of eccentric contraction as a measure of risk associated with various walking conditions and other activities. The outcomes of these future studies will help establish the much needed quantitative guidance for activities or exercises in patients with DMD, as unselectively limiting activities or exercises (such as walking) can be detrimental (Markert et al., 2011; Lovering and Brooks, 2014; Birnkrant et al., 2018).

In addition, our study showed the level of muscle coactivation strongly predicts the amount of muscle eccentric contraction (Figure 5B). During level walking, muscle coactivations in patients with DMD are greater compared to healthy controls and as the disease progresses (Ropars et al., 2016; Rinaldi et al., 2020). Although these previous studies have speculated that the increase of coactivation in patients with DMD is needed to preserve walking stability under muscle weaknesses, the potential of coactivation to lead to adverse increases of eccentric contraction may also need to be carefully studied in future studies. Similar approaches with musculoskeletal simulations can be used to probe the potential connections among muscle weakness, muscle coactivation and muscle eccentric contraction in patients with DMD, which will improve the understanding of disease progression in DMD.

There are some potential limitations of this study that should be acknowledged. First, the walking simulations in the current study were derived from a group of healthy adult participants. Patients with DMD have some aspects of walking kinematics and kinetics that differ from healthy participants (D’Angelo et al., 2009; Thomas et al., 2010; Goudriaan et al., 2018). These differences mainly result from the pathophysiological changes of the musculoskeletal structure in DMD, such as joint contracture (Gaudreault et al., 2009) and muscle weakness due to fibrosis and fatty infiltration (Klingler et al., 2012; Wokke et al., 2014), which may limit the direct extrapolations of some findings in the current study to patients with DMD. However, by investigating sloped walking in healthy participants, the current study provides fundamental biomechanical mechanisms of how sloped walking, as a type of locomotion, alters amounts of muscle eccentric contractions compared to level walking. These mechanisms can lead to further hypothesis-driven studies that improve the understanding and management of DMD.

Second, the current analyses were based on a study that simulated sloped walking on a treadmill (Pickle et al., 2016). Although there may be biomechanical differences between treadmill and overground walking (Riley et al., 2007; Lee and Hidler, 2008), the joint angles and moments estimated in Pickle et al. (2016) were similar to previous studies using overground ramps (Lay et al., 2006; Silverman et al., 2012). Therefore, it is unlikely that our findings would be substantially different if they were based on sloped walking on overground ramps. Third, the coactivations in the current study were calculated from the muscle activations estimated with a efficiency-based cost function in optimization (minimizing the sum of muscle excitations squared) (Thelen and Anderson, 2006). Although the previous study (Pickle et al., 2016) showed that muscle activations predicted by simulations were similar to measured EMG signals, this cost function only employs muscle coactivations that effectively used biarticular muscles to facilitate biomechanical efficiency in the lower limbs (Herzog and Binding, 1992). In addition to efficiency, other mechanisms may also lead to muscle coactivations, such as increasing joint stiffness to maintain stability when walking on slopes (Hortobágyi and DeVita, 2000; Franz and Kram, 2013). When coactivations of agonist-antagonist muscle pairs estimated from simulations were compared to those from experimentally measured EMG signals during sloped walking, there were some differences, but the general trends were largely similar (see the Supplementary Material for details). This indicated that the efficiency may be the primary factor influencing muscle coactivations during sloped walking, in which the demands of joint moments greatly increase (Redfern and Dipasquale, 1997; Lay et al., 2006; Alexander and Schwameder, 2016c). Nevertheless, muscle eccentric contractions will likely increase with muscle coactivation (Figure 5B) regardless of the underlying mechanisms of coactivation.

## Conclusion

Currently, there is no clear consensus on how patients with DMD should participate in various types of activities and/or exercises (frequency, intensity, and duration) (Markert et al., 2011; Lovering and Brooks, 2014). Most existing knowledge regarding the effects of activities and exercises on DMD is from animal studies (Dupont-Versteegden et al., 1994; Hayes and Williams, 1996; Baltgalvis et al., 2012; Selsby et al., 2013). However, results from animal studies may not directly translate to human patients (Hu et al., 2017). The current study provides baseline data and biomechanical understanding of sloped walking that could lead to future hypothesis-driven studies to improve the understanding and management of DMD. The simulation approach demonstrated in the current study can be applied to patients to quantify the amount of eccentric contraction as a measure of risks associated with various types of activities and exercises. We hope that this study could serve as a stepping stone that leads to future studies that help establish guidance for activities and exercises in patients with DMD. The results and approach may also be relevant to other types of muscular dystrophy, such as Becker’s muscular dystrophy, that are susceptible to muscle damage induced by eccentric contractions.

## Data Availability Statement

The raw data supporting the conclusions of this article will be made available by the authors, without undue reservation.

## Ethics Statement

The studies involving human participants were reviewed and approved by Human Subject Institutional Review Board, Department of Veterans Affairs. The patients/participants provided their written informed consent to participate in this study.

## Author Contributions

XH and SB conceived the study. XH recreated the simulations and drafted the manuscript. All authors analyzed and interpreted the results, revised the manuscript, and then approved the final version of the manuscript.

## Conflict of Interest

The authors declare that the research was conducted in the absence of any commercial or financial relationships that could be construed as a potential conflict of interest.

## References

[B1] Alexander N. Schwameder H. (2016a). Comparison of estimated and measured muscle activity during inclined walking. *J. Appl. Biomech.* 32 150–159. 10.1123/jab.2015-2021 26502454

[B2] Alexander N. Schwameder H. (2016b). Effect of sloped walking on lower limb muscle forces. *Gait Posture* 47 62–67. 10.1016/j.gaitpost.2016.03.022 27264405

[B3] Alexander N. Schwameder H. (2016c). Lower limb joint forces during walking on the level and slopes at different inclinations. *Gait Posture* 45 137–142. 10.1016/j.gaitpost.2016.01.022 26979896

[B4] Alexander N. Strutzenberger G. Ameshofer L. M. Schwameder H. (2017). Lower limb joint work and joint work contribution during downhill and uphill walking at different inclinations. *J. Biomech.* 61 75–80. 10.1016/j.jbiomech.2017.07.001 28734544

[B5] Arpan I. Forbes S. C. Lott D. J. Senesac C. R. Daniels M. J. Triplett W. T. (2013). T2 mapping provides multiple approaches for the characterization of muscle involvement in neuromuscular diseases: a cross-sectional study of lower leg muscles in 5-15-year-old boys with Duchenne muscular dystrophy. *NMR Biomed.* 26 320–328. 10.1002/nbm.2851 23044995 PMC3573223

[B6] Baltgalvis K. A. Call J. A. Cochrane G. D. Laker R. C. Yan Z. Lowe D. A. (2012). Exercise training improves plantar flexor muscle function in mdx mice. *Med. Sci. Sports Exerc.* 44 1671–1679. 10.1249/MSS.0b013e31825703f0 22460476 PMC3470762

[B7] Birnkrant D. J. Bushby K. Bann C. M. Apkon S. D. Blackwell A. Brumbaugh D. (2018). Diagnosis and management of Duchenne muscular dystrophy, part 1: diagnosis, and neuromuscular, rehabilitation, endocrine, and gastrointestinal and nutritional management. *Lancet Neurol.* 17 251–267. 10.1016/s1474-4422(18)30024-30023 29395989 PMC5869704

[B8] Childers M. K. Okamura C. S. Bogan D. J. Bogan J. R. Petroski G. F. McDonald K. (2002). Eccentric contraction injury in dystrophic canine muscle. *Archiv. Phys. Med. Rehabil.* 83 1572–1578. 10.1053/apmr.2002.35109 12422328

[B9] D’Angelo M. G. Berti M. Piccinini L. Romei M. Guglieri M. Bonato S. (2009). Gait pattern in Duchenne muscular dystrophy. *Gait Posture* 29 36–41. 10.1016/j.gaitpost.2008.06.002 18656361

[B10] Delp S. L. Anderson F. C. Arnold A. S. Loan P. Habib A. John C. T. (2007). OpenSim: open-source software to create and analyze dynamic simulations of movement. *IEEE Trans. Biomed. Eng.* 54 1940–1950. 10.1109/TBME.2007.901024 18018689

[B11] Delp S. L. Loan J. P. Hoy M. G. Zajac F. E. Topp E. L. Rosen J. M. (1990). An interactive graphics-based model of the lower extremity to study orthopaedic surgical procedures. *Biomed. Eng. IEEE Trans.* 37 757–767. 10.1109/10.102791 2210784

[B12] DeVita P. Helseth J. Hortobagyi T. (2007). Muscles do more positive than negative work in human locomotion. *J. Exp. Biol.* 210(Pt 19), 3361–3373. 10.1242/jeb.003970 17872990 PMC2577758

[B13] Dewolf A. H. Ivanenko Y. Zelik K. E. Lacquaniti F. Willems P. A. (2018). Kinematic patterns while walking on a slope at different speeds. *J. Appl. Physiol.* 125 642–653. 10.1152/japplphysiol.01020.2017 29698109 PMC6842866

[B14] Dupont-Versteegden E. E. McCarter R. J. Katz M. S. (1994). Voluntary exercise decreases progression of muscular dystrophy in diaphragm of mdx mice. *J. Appl. Physiol.* 77 1736–1741. 10.1152/jappl.1994.77.4.1736 7836193

[B15] Farris D. J. Sawicki G. S. (2012). The mechanics and energetics of human walking and running: a joint level perspective. *J. R. Soc. Interf.* 9 110–118. 10.1098/rsif.2011.0182 21613286 PMC3223624

[B16] Franz J. R. Kram R. (2012). The effects of grade and speed on leg muscle activations during walking. *Gait Posture* 35 143–147. 10.1016/j.gaitpost.2011.08.025 21962846 PMC3262943

[B17] Franz J. R. Kram R. (2013). How does age affect leg muscle activity/coactivity during uphill and downhill walking? *Gait Posture* 37 378–384. 10.1016/j.gaitpost.2012.08.004 22940542 PMC3538118

[B18] Franz J. R. Lyddon N. E. Kram R. (2012). Mechanical work performed by the individual legs during uphill and downhill walking. *J. Biomech.* 45 257–262. 10.1016/j.jbiomech.2011.10.034 22099148 PMC3246037

[B19] Gaudreault N. Gravel D. Nadeau S. (2009). Evaluation of plantar flexion contracture contribution during the gait of children with Duchenne muscular dystrophy. *J. Electromyogr. Kinesiol.* 19 e180–e186. 10.1016/j.jelekin.2007.09.004 17977021

[B20] Goudriaan M. Van den Hauwe M. Dekeerle J. Verhelst L. Molenaers G. Goemans N. (2018). Gait deviations in Duchenne muscular dystrophy-Part 1. A systematic review. *Gait Posture* 62 247–261. 10.1016/j.gaitpost.2018.03.020 29579701

[B21] Hayes A. Williams D. A. (1996). Beneficial effects of voluntary wheel running on the properties of dystrophic mouse muscle. *J. Appl. Physiol.* 80 670–679. 10.1152/jappl.1996.80.2.670 8929614

[B22] Herzog W. Binding P. (1992). Predictions of antagonistic muscular activity using nonlinear optimization. *Math. Biosci.* 111 217–229. 10.1016/0025-5564(92)90071-4 1515744

[B23] Holm S. (1979). A simple sequentially rejective multiple test procedure. *Scand. J. Statist.* 6 65–70.

[B24] Hortobágyi T. DeVita P. (2000). Muscle pre- and coactivity during downward stepping are associated with leg stiffness in aging. *J. Electromyogr. Kinesiol.* 10 117–126. 10.1016/s1050-6411(99)00026-27 10699559

[B25] Hu X. Blemker S. S. (2015). Musculoskeletal simulation can help explain selective muscle degeneration in Duchenne muscular dystrophy. *Muscle Nerve* 52 174–182. 10.1002/mus.24607 25704785

[B26] Hu X. Charles J. P. Akay T. Hutchinson J. R. Blemker S. S. (2017). Are mice good models for human neuromuscular disease? Comparing muscle excursions in walking between mice and humans. *Skelet. Muscle* 7:26 10.1186/s13395-017-0143-149 PMC5689180 29145886

[B27] Kimel-Naor S. Gottlieb A. Plotnik M. (2017). The effect of uphill and downhill walking on gait parameters: a self-paced treadmill study. *J. Biomech.* 60 142–149. 10.1016/j.jbiomech.2017.06.030 28757238

[B28] Klingler W. Jurkat-Rott K. Lehmann-Horn F. Schleip R. (2012). The role of fibrosis in Duchenne muscular dystrophy. *Acta Myol.* 31 184–195. 23620650 PMC3631802

[B29] Kuster M. Sakurai S. Wood G. A. (1995). Kinematic and kinetic comparison of downhill and level walking. *Clin. Biomech.* 10 79–84. 10.1016/0268-0033(95)92043-l 11415535

[B30] Lange G. W. Hintermeister R. A. Schlegel T. Dillman C. J. Steadman J. R. (1996). Electromyographic and kinematic analysis of graded treadmill walking and the implications for knee rehabilitation. *J. Orthop. Sports Phys. Ther.* 23 294–301. 10.2519/jospt.1996.23.5.294 8728527

[B31] Lay A. N. Hass C. J. Gregor R. J. (2006). The effects of sloped surfaces on locomotion: a kinematic and kinetic analysis. *J. Biomech.* 39 1621–1628. 10.1016/j.jbiomech.2005.05.005 15990102

[B32] Lay A. N. Hass C. J. Richard Nichols T. Gregor R. J. (2007). The effects of sloped surfaces on locomotion: an electromyographic analysis. *J. Biomech.* 40 1276–1285. 10.1016/j.jbiomech.2006.05.023 16872616

[B33] Le P. Aurand A. Dufour J. S. Knapik G. G. Best T. M. Khan S. N. (2017). Development and testing of a moment-based coactivation index to assess complex dynamic tasks for the lumbar spine. *Clin. Biomech.* 46 23–32. 10.1016/j.clinbiomech.2017.05.001 28500909

[B34] Lee S. J. Hidler J. (2008). Biomechanics of overground vs. treadmill walking in healthy individuals. *J. Appl. Physiol.* 104 747–755. 10.1152/japplphysiol.01380.2006 18048582

[B35] Li W. Zheng Y. Zhang W. Wang Z. Xiao J. Yuan Y. (2015). Progression and variation of fatty infiltration of the thigh muscles in Duchenne muscular dystrophy, a muscle magnetic resonance imaging study. *Neuromuscul. Disord.* 25 375–380. 10.1016/j.nmd.2015.01.003 25701397

[B36] Lovering R. M. Brooks S. V. (2014). Eccentric exercise in aging and diseased skeletal muscle: good or bad? *J. Appl. Physiol.* 116 1439–1445. 10.1152/japplphysiol.00174.2013 23471953 PMC4044401

[B37] Mah J. K. Korngut L. Dykeman J. Day L. Pringsheim T. Jette N. (2014). A systematic review and meta-analysis on the epidemiology of Duchenne and Becker muscular dystrophy. *Neuromuscul. Disord.* 24 482–491. 10.1016/j.nmd.2014.03.008 24780148

[B38] Markert C. D. Ambrosio F. Call J. A. Grange R. W. (2011). Exercise and duchenne muscular dystrophy: toward evidence-based exercise prescription. *Muscle Nerve* 43 464–478. 10.1002/mus.21987 21404285

[B39] Markert C. D. Case L. E. Carter G. T. Furlong P. A. Grange R. W. (2012). Exercise and Duchenne muscular dystrophy: where we have been and where we need to go. *Muscle Nerve* 45 746–751. 10.1002/mus.23244 22499105

[B40] Montgomery J. R. Grabowski A. M. (2018). The contributions of ankle, knee and hip joint work to individual leg work change during uphill and downhill walking over a range of speeds. *R. Soc. Open Sci.* 5:180550. 10.1098/rsos.180550 30225047 PMC6124028

[B41] Petrof B. J. Shrager J. B. Stedman H. H. Kelly A. M. Sweeney H. L. (1993). Dystrophin protects the sarcolemma from stresses developed during muscle contraction. *Proc. Natl. Acad. Sci. U.S.A.* 90 3710–3714. 10.1073/pnas.90.8.3710 8475120 PMC46371

[B42] Pickle N. T. Grabowski A. M. Auyang A. G. Silverman A. K. (2016). The functional roles of muscles during sloped walking. *J. Biomech.* 49 3244–3251. 10.1016/j.jbiomech.2016.08.004 27553849 PMC5167499

[B43] Prilutsky B. I. (2000). Coordination of two- and one-joint muscles: functional consequences and implications for motor control. *Mot. Control* 4 1–44. 10.1123/mcj.4.1.1 10675807

[B44] Redfern M. S. Dipasquale J. (1997). Biomechanics of descending ramps. *Gait Posture* 6 119–125. 10.1016/s0966-6362(97)01117-x

[B45] Riley P. O. Paolini G. Della Croce U. Paylo K. W. Kerrigan D. C. (2007). A kinematic and kinetic comparison of overground and treadmill walking in healthy subjects. *Gait Posture* 26 17–24. 10.1016/j.gaitpost.2006.07.003 16905322

[B46] Rinaldi M. Petrarca M. Romano A. Vasco G. D’Anna C. Bibbo D. (2020). Progression of muscular co-activation and gait variability in children with Duchenne muscular dystrophy: A 2-year follow-up study. *Clin. Biomech.* 78:105101. 10.1016/j.clinbiomech.2020.105101 32652381

[B47] Ropars J. Lempereur M. Vuillerot C. Tiffreau V. Peudenier S. Cuisset J. M. (2016). Muscle activation during gait in children with Duchenne muscular dystrophy. *PLoS One* 11:e0161938. 10.1371/journal.pone.0161938 27622734 PMC5021331

[B48] Selsby J. T. Acosta P. Sleeper M. M. Barton E. R. Sweeney H. L. (2013). Long-term wheel running compromises diaphragm function but improves cardiac and plantarflexor function in the mdx mouse. *J. Appl. Physiol.* 115 660–666. 10.1152/japplphysiol.00252.2013 23823150 PMC3763072

[B49] Silverman A. K. Wilken J. M. Sinitski E. H. Neptune R. R. (2012). Whole-body angular momentum in incline and decline walking. *J. Biomech.* 45 965–971. 10.1016/j.jbiomech.2012.01.012 22325978

[B50] Spaulding H. R. Selsby J. T. (2018). Is exercise the right medicine for dystrophic muscle? *Med. Sci. Sports Exerc.* 50 1723–1732. 10.1249/mss.0000000000001639 29649068

[B51] Thelen D. Anderson F. (2006). Using computed muscle control to generate forward dynamic simulations of human walking from experimental data. *J. Biomech.* 39 1107–1115. 10.1016/j.jbiomech.2005.02.010 16023125

[B52] Thomas S. S. Buckon C. E. Nicorici A. Bagley A. McDonald C. M. Sussman M. D. (2010). Classification of the gait patterns of boys with Duchenne muscular dystrophy and their relationship to function. *J. Child Neurol.* 25 1103–1109. 10.1177/0883073810371002 20587736 PMC3794706

[B53] Willcocks R. J. Arpan I. A. Forbes S. C. Lott D. J. Senesac C. R. Senesac E. (2014). Longitudinal measurements of MRI-T2 in boys with Duchenne muscular dystrophy: effects of age and disease progression. *Neuromuscul. Disord.* 24 393–401. 10.1016/j.nmd.2013.12.012 24491484 PMC4277599

[B54] Winter B. (2013). Linear models and linear mixed effects models in R with linguistic applications. *arXiv* [Preprint], Available online at: https://arxiv.org/abs/1308.5499 (accessed September 28, 2020).

[B55] Wokke B. H. van den Bergen J. C. Versluis M. J. Niks E. H. Milles J. Webb A. G. (2014). Quantitative MRI and strength measurements in the assessment of muscle quality in Duchenne muscular dystrophy. *Neuromusc. Disord.* 24 409–416. 10.1016/j.nmd.2014.01.015 24613733

