# Supplementary material for: Muscle Eccentric Contractions Increase in Downhill and High-Grade Uphill Walking
Source: Front Bioeng Biotechnol. 2020 Oct 14;8:573666. doi: 10.3389/fbioe.2020.573666 (PMC7591807; doi:10.3389/fbioe.2020.573666)
Supplement: Supplementary file 1 [file Data_Sheet_1.docx]

**Supplementary Material**

# Supplement Figures


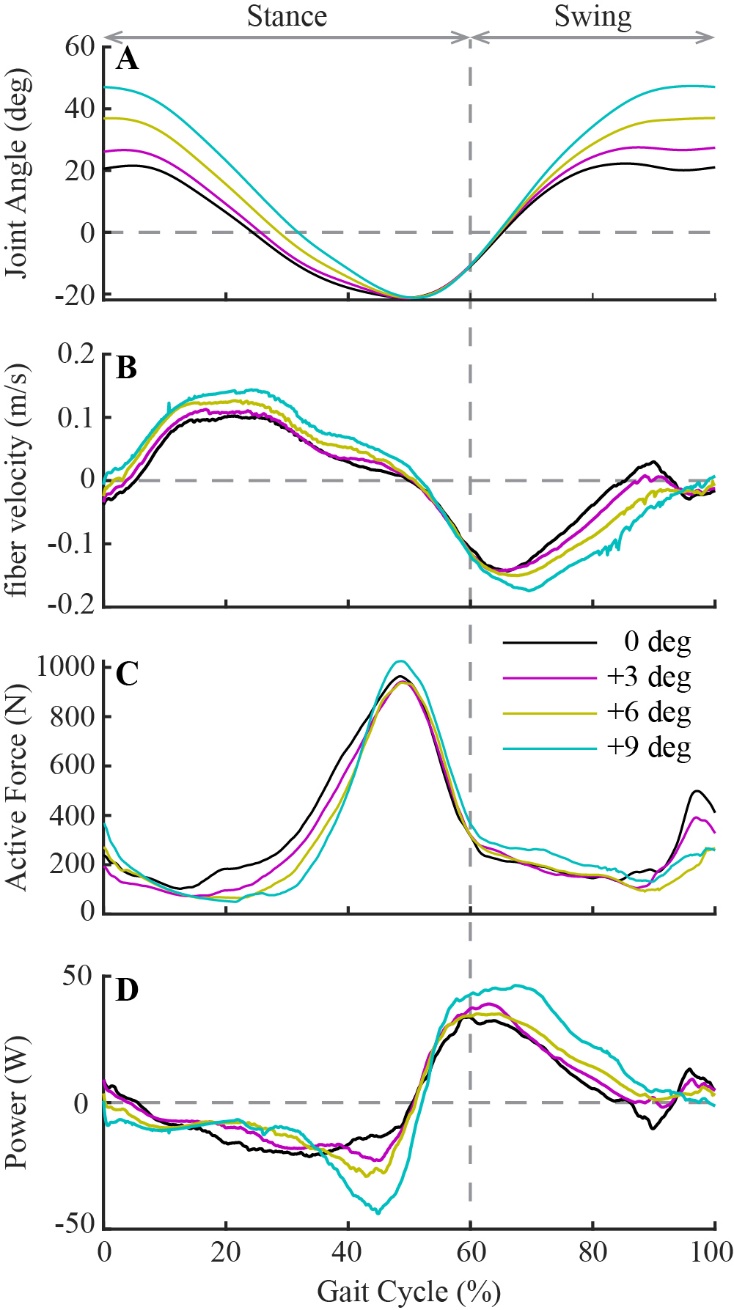


**Supplement Figure 1.** The comparisons of mean (across all participants) hip flexion/extension angle (A; flexion is positive) and iliacus fiber velocity (B; lengthening is positive), active force (C) and active fiber power (D) between uphill and level walking in one gait cycle. Note in the range of 40-50% of the gait cycle, although active force of iliacus is lower in uphill (+9°) than in level (0°) walking, the negative power of iliacus is greater in uphill than in level walking due to higher lengthening velocity in uphill walking.


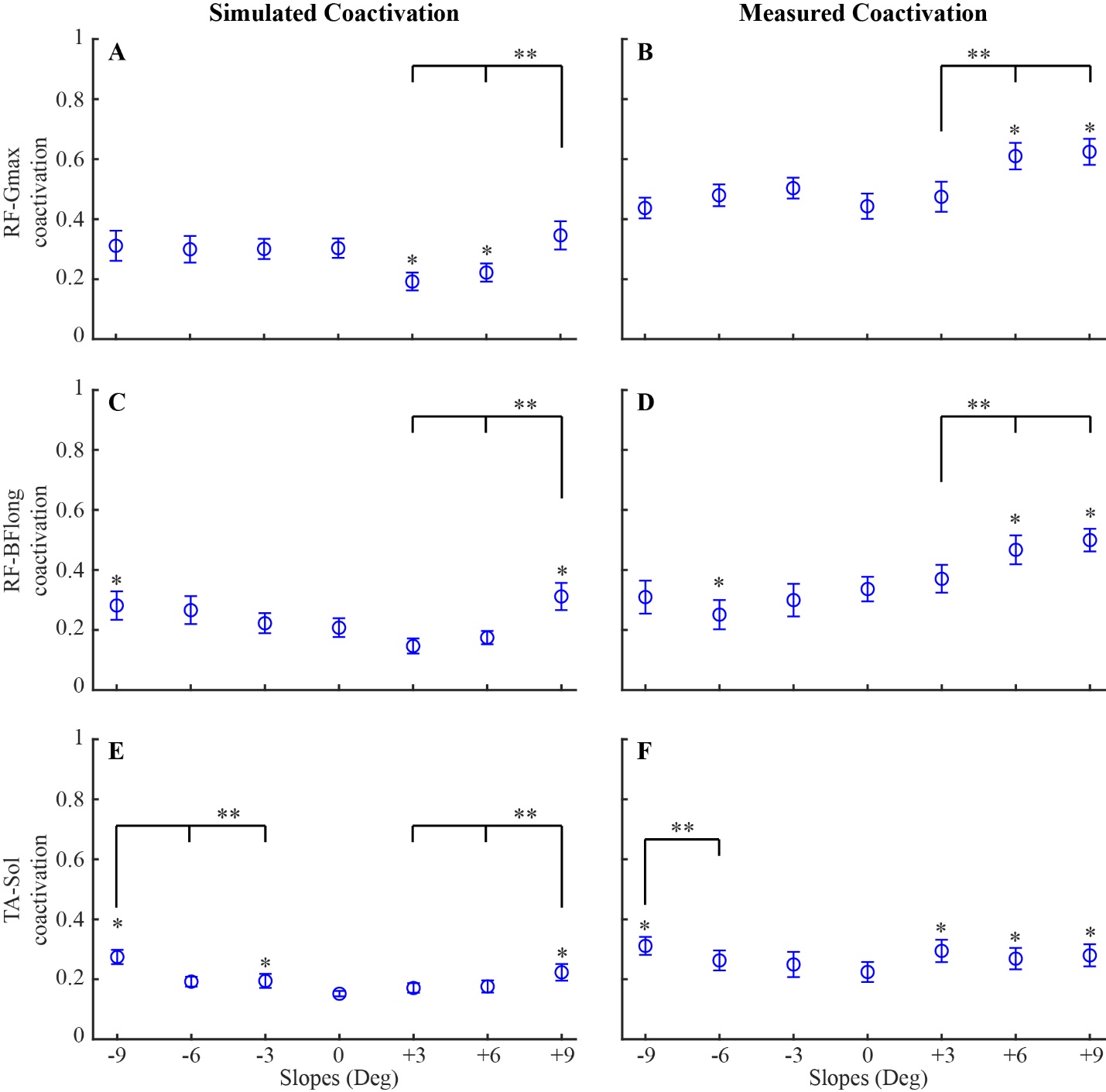


**Supplement Figure 2.** The coactivation indices (CI) of agonist-antagonist muscle pairs in sloped and level walking (mean and standard error across participants; details on the next page). Left column (A, C, and E) is CIs based on simulated muscle activations, and the right column (B, D, and F) is CIs based on measured muscle activations. ’*’= significant difference from level walking (*p* < 0.05). ‘**’ = significant difference between compared pairs of sloped walking (*p* < 0.05).

# Coactivations of agonist-antagonist muscle pairs

Coactivations of agonist-antagonist muscle pairs were assessed based on the EMG signals from experiments and muscle activations from walking simulations. The EMG signals from the gluteus maximus (Gmax), biceps femoris long head (BFlong), rectus femoris (RF), soleus (Sol) and tibialis anterior (TA) were previously recorded and processed (mean-removed, rectified, low-pass filtered and normalized) in (Pickle et al., 2016). The same study (Pickle et al., 2016) also estimated the activations of lower limb muscles in the development of the dynamic simulations of sloped walking, from which the activations of five corresponding muscles were extracted for analyses in the current study. The experimentally measured and simulated activation envelopes of Gmax, BFlong, RF, Sol and TA were used to calculate the coactivation indices for agonist-antagonist muscle pairs (*CI_mus_*) of RF-Gmax, RF-BFlong and TA-Sol over a gait cycle as in (Franz and Kram, 2013):

${CI}_{mus}\left( {act}_{1}, {act}_{2} \right)=2*\left( \frac{\int\text{min}\left( {act}_{1}, {act}_{2} \right)}{\int\text{min}\left( {act}_{1}, {act}_{2} \right)+\int\text{max}\left( {act}_{1}, {act}_{2} \right)} \right)$ (1)

where min and max represent the minimum and maximum of the activation envelopes of two muscles (*act*_1_ and *act*_2_) at each time step of a gait cycle, respectively. The *CI_mus_* of measured and simulated muscle activations was calculated separately. Similar linear mixed-effects models described in the Methods were used to examine the effects of slopes on muscle coactivations.

Comparing the coactivations of muscle pairs calculated based on simulation-predicted activations to those based on experimentally measured activations generally showed similar trends, but there were also some differences. In uphill walking, the coactivations of RF-Gmax and RF-BFlong were largely comparable to or greater than in level walking, consistent with previous findings (Franz and Kram, 2013). The only exceptions were that the simulation-predicted coactivations of RF-Gmax at +3° (*p* < 0.001) and +6° (*p* < 0.001) were lower compared to 0° (Supplement Fig. 2A-D). However, the simulation-predicted coactivation of RF-Gmax increased at +9° compared to +3° (*p* < 0.001) and +6° (*p* < 0.001) and became comparable to 0° (*p* = 0.37). In general, coactivations of RF-Gmax and RF-BFlong largely increased with the slope grades in uphill walking: all simulated and measured coactivations at +9° were higher than those at +3° (*p* < 0.05). In downhill walking, the coactivations of RF-Gmax and RF-BFlong were also largely comparable to or higher than those in level walking, only except for measured BFlong-RF at -6° (*p* = 0.003; Supplement Fig. 2A-D). In addition, across the three grades of downhill slopes, the coactivations of RF-Gmax and RF-BFlong were not different in all simulated and measured cases. Generally, although the coactivations of TA-Sol increased in sloped walking compared to level walking, in both simulated and measured cases, the magnitudes of the differences were small across six slopes compared to those of RF-Gmax and RF-BFlong (Supplement Fig. 2E and F).

# References

Franz, J.R., and Kram, R. (2013). How does age affect leg muscle activity/coactivity during uphill and downhill walking? *Gait Posture* 37(3)**,** 378-384. doi: 10.1016/j.gaitpost.2012.08.004.

Pickle, N.T., Grabowski, A.M., Auyang, A.G., and Silverman, A.K. (2016). The functional roles of muscles during sloped walking. *J Biomech* 49(14)**,** 3244-3251. doi: 10.1016/j.jbiomech.2016.08.004.
